# Modified Characteristic Finite Element Method with Second-Order Spatial Accuracy for Solving Convection-Dominated Problem on Surfaces

**DOI:** 10.3390/e25121631

**Published:** 2023-12-07

**Authors:** Longyuan Wu, Xinlong Feng, Yinnian He

**Affiliations:** 1 College of Mathematics and Systems Science, Xinjiang University, Urumqi 830017, China; wly2329@foxmail.com (L.W.); fxlmath@xju.edu.cn (X.F.); 2 School of Mathematics and Statistics, Xi’an Jiaotong University, Xi’an 710049, China

**Keywords:** surface convection–reaction–diffusion equations, explicit–implicit method, Taylor expansion, surface finite element, stability

## Abstract

We present a modified characteristic finite element method that exhibits second-order spatial accuracy for solving convection–reaction–diffusion equations on surfaces. The temporal direction adopted the backward-Euler method, while the spatial direction employed the surface finite element method. In contrast to regular domains, it is observed that the point in the characteristic direction traverses the surface only once within a brief time. Thus, good approximation of the solution in the characteristic direction holds significant importance for the numerical scheme. In this regard, Taylor expansion is employed to reconstruct the solution beyond the surface in the characteristic direction. The stability of our scheme is then proved. A comparison is carried out with an existing characteristic finite element method based on face mesh. Numerical examples are provided to validate the effectiveness of our proposed method.

## 1. Introduction

The convection–reaction–diffusion (CRD) equation holds significant importance in the field of fluid mechanics as it serves as a model that captures the interconnected processes of convection, reaction, and diffusion. This equation proves instrumental in elucidating various natural phenomena such as alterations in liquid pollutants upon discharge into rivers, the durability of reinforced concrete in seawater [1], and heat conduction, among others. However, certain practical phenomena frequently manifest in irregular domains, including biological films, pattern formation [2,3], surfactant transportation [4], evolution of colonies on irregular surfaces [5], and cell migration [6,7]. The governing equation of these phenomena is the CRD equation on surfaces, thus necessitating the exploration of numerical methods for solving these equations on surfaces.

The numerical methods for solving partial differential Equations (PDEs) on surfaces can be divided into two main categories: mesh-free methods [8,9,10,11,12] and mesh-based methods [13,14,15,16,17,18,19,20,21,22,23,24,25,26,27,28,29,30]. For mesh-free methods, the implementation of this particular method is relatively straightforward. However, there are challenges in stability and error estimation. Conversely, mesh-based methods are highly dependent on the mesh, but improve the stability of the numerical method. We focus here on the finite element method which is one of the mesh-based methods for solving PDEs on surfaces. The mesh generation include two prevalent strategies: embedding the surface in the narrow-band domain [16,17,18,19,20,21,22,23] and directly discretizing the surface [25,26,27,28,29,30].

When convection is dominant, the effectiveness of classical finite element methods is reduced, yielding non-physical oscillations in the numerical solution. To alleviate these oscillations, various finite element methods have been introduced, such as Olshanskii et al. [22], who have presented error estimates for a trace finite element method with streamline-upwind/Petrov–Galerkin stabilization. This represents the first residual-based stabilization method for convection–diffusion equations on surfaces. However, the accuracy and stability of this method depend on the careful selection of the stabilization parameters, which requires rigorous theoretical considerations. For the same problem, Simon and Tobiska provide a priori error estimation for a fitted finite element local projection stabilization in their study, which is symmetric stability terms [26]. Recently, Xiao et al. developed gradient recovery-based adaptive techniques in [27]. Jin et al. [28] subsequently utilized their work [27] to address the convection-dominated problem on surfaces using mixed finite element methods and provided theoretical analysis.

The characteristic finite element method [23,29,31] is easier to operate than the above methods [22,26,27,28]. To implement this method, it is necessary to convert the CRD equation into a reaction–diffusion equation and interpolate the solution in the characteristic direction [31]. Unlike the regular domains [31], the point in the characteristic direction pass through the surface only once during a short time. Consequently, the solution situated beyond the surface in the characteristic direction needs to be reconstructed based on available information. This task is straightforward for the characteristic finite element method based on volume mesh [23]. However, it presents challenges for the face mesh-based method in Section 4 of [25]. In response, Xiao et al. [29] introduced a characteristic finite element method (CFEM) that relies on the face mesh. By incorporating mass lumping, CFEM ensures the preservation of positivity, albeit at the cost of diminished accuracy. Here, we will propose a modified characteristic finite element method with second-order spatial accuracy for solving the CRD equation on surfaces. The temporal discretization strategy employed is the backward-Euler method, while the spatial mesh adopts the face mesh method. To reconstruct the solution beyond the surface in the characteristic direction, we consider the Taylor expansion. However, the high accuracy is concomitant with a reduction in the stability. This will result in the transformation of our characteristic finite element method into an explicit–implicit method, thereby compromising its ability to maintain positivity. Then, the reasons for the deterioratation in spatial accuracy of the characteristic finite element method based on face mesh are analyzed.

The rest of this paper is organized as follows. We start, in Section 2, with an introduction to differential operators, Green’s theorem on surface and the CRD equation on surfaces. The reaction–diffusion equation with a characteristic directional derivative is then given. Additionally, we propose a modified characteristic finite element method based on Taylor expansion in Section 3. Then, the reconstruction methods proposed by us and the CFEM are examined. Lastly, in Section 4, we present a series of numerical examples to assess the disparities between our scheme and the CFEM. The conclusion of our findings is provided in Section 5.

## 2. Preliminaries

This section aims to present a thorough introduction to surface operators, Green’s theorem on surface and the CRD equation involved. Subsequently, the CRD equation is converted into a reaction–diffusion equation with a characteristic directional derivative employing a characteristic finite element method.

### 2.1. Surface Operators and Green’s Theorem on Surface

Let $\Gamma =\{x\in R^{3}|\;\psi \left(x\right)=0\}$ be a connected and oriented surface without boundary for such $\psi \in C^{2}\left(R^{3}\right)$. For x∈Γ, $P\left(x\right)=I-n\left(x\right)n^{T}\left(x\right)$ denotes the tangential projection operator where $n\left(x\right)=\frac{\nabla \psi (x)}{\left|\nabla \psi (x)\right|}$ in the unit vector normal to Γ. The tangential gradient of $f\in C^{2}\left(\Gamma \right)$ is obtained with

(1) $$\nabla_{\Gamma }f\left(x\right)=P\left(x\right)\cdot \nabla f\left(x\right)\;x\in \Gamma ,$$

and the Laplace–Beltremi operator is defined as

(2) $$\Delta_{\Gamma }f\left(x\right)=\nabla_{\Gamma }\cdot \nabla_{\Gamma }f\left(x\right)\;f\left(x\right)\in C^{2}\left(\Gamma \right).$$

Let *H* be the mean curvature of Γ; Green’s theorem on any hypersurface is as follows.

**Theorem** **1** ([25])**.** *If the boundary ∂Γ of a hypersurface $\Gamma \subset R^{n+1},n=1,2,\ldots ,$ is smooth, then $f\in C^{1}\left(\bar{\Gamma }\right)$ satisfy*

$$\int_{\Gamma }\nabla_{\Gamma }fdA=\int_{\Gamma }HfndA+\int_{\partial \Gamma }f\nu d\sigma ,$$

*where ν is the co-normal of *Γ*, and dA and dσ are the measures of Γ and ∂Γ, respectively.*

By choosing the inner product ${\left(u,v\right)}_{\Gamma }=\int_{\Gamma }uvdA$, the norms $\left|\right|\cdot {\left|\right|}_{L^{2}\left(\Gamma \right)}$ and $\left|\right|\cdot {\left|\right|}_{H^{1}\left(\Gamma \right)}$ are defined with

(3) $${\left||u|\right|}_{L^{2}\left(\Gamma \right)}=\sqrt{{\left(u,u\right)}_{\Gamma }}{\;||u||}_{H^{1}\left(\Gamma \right)}^{2}={\left||u|\right|}_{L^{2}\left(\Gamma \right)}^{2}+\left|\right|\nabla_{\Gamma }u{\left|\right|}_{L^{2}\left(\Gamma \right)}^{2}.$$

### 2.2. The Convection–Reaction–Diffusion Equations on Surface

In this paper, we will focus on the following equation:

(4) $$\left\{\begin{array}{cc} \partial_{t}u+\beta \cdot \nabla_{\Gamma }u-\epsilon \Delta_{\Gamma }u+\mu u=f & (x,t)\in \Gamma \times (0,T],\\ u\left(x,0\right)=u_{0}\left(x\right) & x\in \Gamma , \end{array}\right.$$

where the diffusion parameter ϵ and the reaction coefficient μ are positive constants. The convection velocity β are assumed to be time-independent and continuous function satisfying

(5) $$\mu -\frac{1}{2}\nabla_{\Gamma }\cdot \beta_{\Gamma }\geq \kappa >0.$$

According to orthogonality, the convection term in (4) can be modified as

(6) $$\beta \cdot \nabla_{\Gamma }u=\beta_{\Gamma }\cdot \nabla_{\Gamma }u=\beta_{\Gamma }\cdot \left(P\nabla \right)u=\beta_{\Gamma }\cdot \left(I-P+P\right)\nabla u=\beta_{\Gamma }\cdot \nabla u,$$

where $\beta_{\Gamma }$ represents the projection of β onto the tangent plane of Γ. This means that we only need to pay attention to the tangential component $\beta_{\Gamma }$ of β at the surface Γ.

### 2.3. The Reaction–Diffusion Equation with Characteristic Directional Derivative

For a small positive parameter δ, let U(Γ,δ) be the neighbourhood of the surface Γ and $t^{n}=n\Delta t,n=0,1,\ldots ,N$, and parameter Δt=T/N is the time step with Δt≤δ. We extend $u(x,t^{n})$ from surface Γ to neighborhood U(Γ,δ). Assume that there is a characteristic direction τ in $U\left(\Gamma ,\delta \right)\times \left[t^{n-1},t^{n}\right]$ and the point (χ(t),t) on direction τ satisfy

(7) $$\left\{\begin{array}{c} \frac{d}{dt}\chi \left(t\right)=\beta_{\Gamma }\;t\in \left[t^{n-1},t^{n}\right),\\ x=\chi (t^{n}). \end{array}\right.$$

Integrating the characteristic Equation (7) over $\left[t^{n-1},t^{n}\right]$, the backtracking characteristic point obtained is as follows:

(8) $$\chi \left(t^{n-1}\right)=x-\Delta t\beta_{\Gamma }\equiv \overset{ˇ}{x}.$$

Thanks to (7), the characteristic directional derivative at point (χ(t),t) is

(9) $$\partial_{\tau }u=\frac{d}{dt}u\left(\chi \left(t\right),t\right)=\partial_{t}u+\nabla u\cdot \frac{d}{dt}\chi \left(t\right)=\partial_{t}u+\beta_{\Gamma }\cdot \nabla u.$$

It follows from (4) and (9) that

(10) $$\left\{\begin{array}{cc} \partial_{\tau }u-\varepsilon \Delta_{\Gamma }u+\mu u=f & (x,t)\in \Gamma \times (0,T],\\ u\left(x,0\right)=u_{0}\left(x\right) & x\in \Gamma . \end{array}\right.$$

Several discrete schemes of the characteristic directional derivative $\partial_{\tau }u\left(t^{n}\right)$ are known, including the backward-Euler method, the Crank–Nicolson method, and the Runge–Kutta method. In this paper, we employ the backward-Euler scheme:

(11) $$\partial_{\tau }u\left(x,t^{n}\right)=\frac{u\left(x,t^{n}\right)-u\left(\overset{ˇ}{x},t^{n-1}\right)}{\Delta t}+O\left(\Delta t\right).$$

The backtracking characteristic point $\overset{ˇ}{x}$ is evidently not situated on Γ. Consequently, it is of utmost significance to reconstruct $u(\overset{ˇ}{x},t^{n-1})$ by utilizing the available information on surfaces.

## 3. A Modified Characteristic Finite Element Method (MCFEM) Based on Taylor Expansion

In this section, we will introduce a modified characteristic finite element method (MCFEM) which employs the Taylor expansion to obtain the solution beyond the surface in the characteristic direction. Then, we will provide a specific discrete formulation of the MCFEM and analyze its stability.

### 3.1. The Reconstruction Method Based on Taylor Expansion

The approximation of the solution in the characteristic direction is heavily mesh-dependent. If the volume mesh [16,17,18,19,20,21,22,23] is used, the solution beyond the surface can be reconstructed using interpolation. However, the face mesh in Section 4 of [25] cannot generate a narrow band which contains surfaces similar to the volume mesh. Consequently, if a point in the characteristic direction is situated on the current mesh, it must have resided beyond the mesh at the previous moment. This presents novel challenges to the reconstruction method.

**Figure 1:**
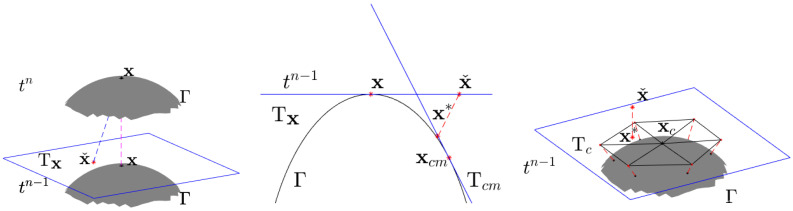
The schematic diagram of the CFEM in [29] for approximate $u(\overset{ˇ}{x})$.

At present, the reconstruction method based on face mesh is only used by Xiao et al. [29], as illustrated in Figure 1. They will identify the nearest mesh point $x_{c}$ and project $\overset{ˇ}{x}$ vertically onto the tangent plane $T_{c}$ of $x_{c}$ as $x^{*}$. Subsequently, the mesh points on each element containing $x_{c}$ are extended into the tangent plane $T_{c}$ along their normal direction to form a new local element. Linear interpolation is then employed to approximate $u(x^{*})$ within these local elements, which is used to approximate $u(\overset{ˇ}{x})$. It is evident that the CFEM in [29] fails to account for the discrepancy between $u(x^{*})$ and $u(\overset{ˇ}{x})$, resulting in a spatial accuracy lower than the second order.

We will suggest a more accurate method for reconstructing the solution beyond the surface. It should be noted that the backtracking characteristic point $\overset{ˇ}{x}$ can be situated within the tangent plane $T_{x}$ of point x∈Γ through the selection of the suitable characteristic direction τ. Considering the Taylor expansion within the tangent plane $T_{x}$ and (8), we have

(12) $$\begin{array}{cc} u(\overset{ˇ}{x},t)\; & =u\left(x,t\right)+\left(\overset{ˇ}{x}-x\right)\cdot \nabla u\left(x,t\right)+O(|\overset{ˇ}{x}-x{|}^{2})\\ \; & =u\left(x,t\right)-\Delta t\beta_{\Gamma }\left(x\right)\cdot \nabla u\left(x,t\right)+O\left(\Delta t^{2}\right)\\ \; & =\overset{ˇ}{\tilde{u}}\left(x,t\right)+O\left(\Delta t^{2}\right), \end{array}$$

where $\overset{ˇ}{\tilde{u}}\left(x\right)$ is an approximation of $u(\overset{ˇ}{x})$ with second-order accuracy in time.

**Figure 2:**
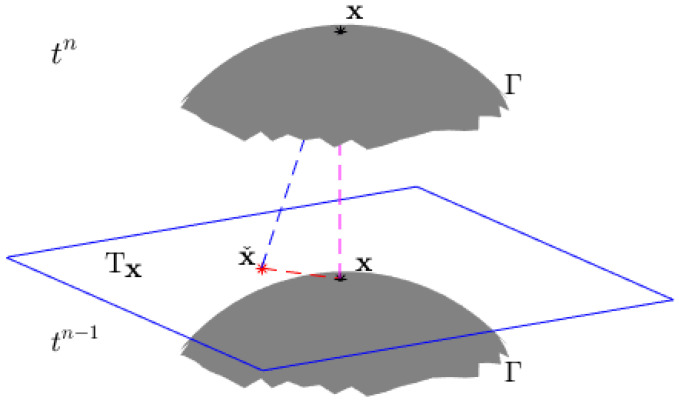
The schematic diagram of our MCFEM for approximate $u(\overset{ˇ}{x})$.

Taking this approximation, we obtain the reconstruction method based on Taylor expansion, as shown in Figure 2. First, the backtracking characteristic point $\left(\overset{ˇ}{x},t^{n-1}\right)$ of point $\left(x,t^{n}\right)$ on Γ is found along the characteristic direction τ. Due to (8), it is easy to know that the backtracking characteristic point is obtained with $\overset{ˇ}{x}=x-\Delta t\beta_{\Gamma }\left(x\right)$. Second, the reconstructed function $\overset{ˇ}{\tilde{u}}\left(x,t^{n-1}\right)=u\left(x,t^{n-1}\right)-\Delta t\beta_{\Gamma }\left(x\right)\cdot \nabla u\left(x,t^{n-1}\right)$ is obtained using the Taylor expansion of $u(\overset{ˇ}{x},t^{n-1})$ at point $\left(x,t^{n-1}\right)$.

### 3.2. Temporal Discretization of the MCFEM

Let ${\bar{\partial }}_{\tau }u\left(t^{n}\right)=\frac{u\left(t^{n}\right)-\overset{ˇ}{\tilde{u}}\left(t^{n-1}\right)}{\Delta t}$ be an approximation of the characteristic directional derivative $\partial_{\tau }u\left(t^{n}\right)$. An estimate between $\partial_{\tau }u\left(t^{n}\right)$ and ${\bar{\partial }}_{\tau }u\left(t^{n}\right)$ is provided below.

**Theorem** **2.** *If $u\in C^{2}\left(U\left(\Gamma ,\delta \right)\times \left[0,T\right]\right)$ satisfies Equation (7), then the following estimate holds:*

(13) $$\left|\right|\partial_{\tau }u\left(t^{n}\right)-{\bar{\partial }}_{\tau }u\left(t^{n}\right){\left|\right|}_{L^{2}\left(\Gamma \right)}\leq \int_{t^{n-1}}^{t^{n}}\left|\right|\partial_{tt}{u||}_{L^{2}\left(\Gamma \right)}+\left|\right|\partial_{t}\left(\partial_{\tau }u\right){\left|\right|}_{L^{2}\left(\Gamma \right)}dt,\;1\leq n\leq N.$$

**Proof.**  By the definition of operator $\partial_{\tau }u\left(t^{n}\right)$ and ${\bar{\partial }}_{\tau }u\left(t^{n}\right)$

(14) $$\begin{array}{cc} \; & \;\left|\right|\partial_{\tau }u\left(t^{n}\right)-{\bar{\partial }}_{\tau }u\left(t^{n}\right){\left|\right|}_{L^{2}\left(\Gamma \right)}\\ \; & =\frac{1}{\Delta t}||\Delta t\left(\partial_{t}u\left(t^{n}\right)+\beta_{\Gamma }\cdot \nabla u\left(t^{n}\right)\right)-\left(u\left(t^{n}\right)-u\left(t^{n-1}\right)+\Delta t\beta_{\Gamma }\cdot \nabla u\left(t^{n-1}\right)\right){\left|\right|}_{L^{2}\left(\Gamma \right)}\\ \; & =\frac{1}{\Delta t}\left|\right|\left[\Delta t\partial_{t}u\left(t^{n}\right)-\left(u\left(t^{n}\right)-u\left(t^{n-1}\right)\right)\right]+\left[\Delta t\beta_{\Gamma }\cdot \nabla \left(u\left(t^{n}\right)-u\left(t^{n-1}\right)\right)\right]{\left|\right|}_{L^{2}\left(\Gamma \right)}\\ \; & :=\frac{1}{\Delta t}\left|\right|T_{1}+T_{2}{\left|\right|}_{L^{2}\left(\Gamma \right)}. \end{array}$$

For $T_{1}$, we see that

(15) $$\begin{array}{cc} T_{1} & =\Delta t\partial_{t}u\left(t^{n}\right)-\left(u\left(t^{n}\right)-u\left(t^{n-1}\right)\right)\\ \; & =\int_{t^{n-1}}^{t^{n}}\partial_{t}u\left(t^{n}\right)-\partial_{t}u\left(t\right)dt\\ \; & =\left(t-t^{n-1}\right)\left(\partial_{t}u\left(t^{n}\right)-\partial_{t}u\left(t\right)\right){|}_{t^{n-1}}^{t^{n}}+\int_{t^{n-1}}^{t^{n}}\left(t-t^{n-1}\right)\partial_{tt}u\left(t\right)dt\\ \; & =\int_{t^{n-1}}^{t^{n}}\left(t-t^{n-1}\right)\partial_{tt}udt. \end{array}$$

For $T_{2}$, we have

(16) $$\begin{array}{cc} T_{2} & =\Delta t\beta_{\Gamma }\cdot \nabla \left(u\left(t^{n}\right)-u\left(t^{n-1}\right)\right)\\ \; & =\Delta t\beta_{\Gamma }\cdot \nabla \left(\int_{t^{n-1}}^{t^{n}}\partial_{t}udt\right)\\ \; & =\Delta t\int_{t^{n-1}}^{t^{n}}\beta_{\Gamma }\cdot \nabla \left(\partial_{t}u\right)dt. \end{array}$$

By substituting (15) and (16) into (14), the bound of $|\partial_{\tau }u\left(t^{n}\right)-{\bar{\partial }}_{\tau }u\left(t^{n}\right)|$ can be obtained with

(17) $$\begin{array}{cc} \; & \;\left|\right|\partial_{\tau }u\left(t^{n}\right)-\frac{u\left(t^{n}\right)-\overset{ˇ}{\tilde{u}}\left(t^{n-1}\right)}{\Delta t}{\left|\right|}_{L^{2}\left(\Gamma \right)}\\ \; & =\frac{1}{\Delta t}\left|\right|\int_{t^{n-1}}^{t^{n}}\left(t-t^{n-1}\right)\partial_{tt}u+\Delta t\beta_{\Gamma }\cdot \nabla \left(\partial_{t}u\right)dt{\left|\right|}_{L^{2}\left(\Gamma \right)}\\ \; & =\frac{1}{\Delta t}\left|\right|\int_{t^{n-1}}^{t^{n}}\left(t-t^{n}\right)\partial_{tt}u+\Delta t\left(\partial_{tt}u+\beta_{\Gamma }\cdot \nabla \left(\partial_{t}u\right)\right)dt\left|\right|\\ \; & =\frac{1}{\Delta t}\left|\right|\int_{t^{n-1}}^{t^{n}}\left(t-t^{n}\right)\partial_{tt}u+\Delta t\partial_{t}\left(\partial_{\tau }u\right)dt{\left|\right|}_{L^{2}\left(\Gamma \right)}\\ \; & \leq \int_{t^{n-1}}^{t^{n}}\left|\right|\partial_{tt}u||+\left|\right|\partial_{t}\left(\partial_{\tau }u\right){\left|\right|}_{L^{2}\left(\Gamma \right)}dt. \end{array}$$

This completes the proof. □

Let $u^{n}$ be an approximation of $u(t^{n})$ and ${\overset{ˇ}{\tilde{u}}}^{n-1}=u^{n-1}-\Delta t\beta_{\Gamma }\cdot \nabla u^{n-1}$. The temporal discretization of (10) is to find $u^{n}\in H^{1}\left(\Gamma \right),n=1,2,\ldots ,N$, recursively

(18) $$\left\{\begin{array}{c} {\left(\bar{\partial_{\tau }}u^{n},v\right)}_{\Gamma }+\epsilon {\left(\nabla_{\Gamma }u^{n},\nabla_{\Gamma }v\right)}_{\Gamma }+{\left(\mu u^{n},v\right)}_{\Gamma }={\left(f^{n},v\right)}_{\Gamma }\;\forall vs.\in H^{1}\left(\Gamma \right),\\ u^{0}=u_{0}, \end{array}\right.$$

where $f^{n}=\frac{1}{\Delta t}\int_{t^{n-1}}^{t^{n}}f\left(t\right)dt$. The stability of the problem (18) is demonstrated in the following manner.

**Theorem** **3.** *Suppose that ${\left\{u^{n}\right\}}_{n=1,2,\ldots ,N}\subset H^{1}\left(\Gamma \right)$ satisfy (18). If $\Delta t\leq \frac{2\epsilon }{\left|\right|\beta_{\Gamma }{\left|\right|}_{L^{2}\left(\Gamma \right)}^{2}}$, then the following inequality holds:*

(19) $$\begin{array}{c} \max\left\{\left|\right|u^{n}-\Delta t\beta_{\Gamma }\cdot \nabla_{\Gamma }u^{n}{\left|\right|}_{L^{2}\left(\Gamma \right)}^{2},\;||u^{n}{\left|\right|}_{L^{2}\left(\Gamma \right)}^{2}+2\Delta t\mu ||u^{n}{\left|\right|}_{L^{2}\left(\Gamma \right)}^{2}+2\Delta t\epsilon ||\nabla_{\Gamma }u^{n}{\left|\right|}_{L^{2}\left(\Gamma \right)}^{2}\right\}\\ \leq e^{T}\left(\left|\right|u^{0}-\Delta t\beta_{\Gamma }\cdot \nabla_{\Gamma }u^{0}{\left|\right|}_{L^{2}\left(\Gamma \right)}^{2}+\int_{0}^{T}{\left||f\left(t\right)|\right|}_{L^{2}\left(\Gamma \right)}^{2}dt\right), \end{array}$$

*for all n.*

**Proof.**  By the definition of $\bar{\partial_{\tau }}u^{n}$, (18) can be written as

(20) $${\left(\frac{u^{n}-\left[u^{n-1}-\Delta t\beta_{\Gamma }\cdot \nabla_{\Gamma }u^{n-1}\right]}{\Delta t},v\right)}_{\Gamma }+\epsilon {\left(\nabla_{\Gamma }u^{n},\nabla_{\Gamma }v\right)}_{\Gamma }+\mu {\left(u^{n},v\right)}_{\Gamma }={\left(f^{n},v\right)}_{\Gamma }.$$

Taking $v=2\Delta tu^{n}$ into (20), we have

(21) $$\begin{array}{cc} \; & \;{\left(2u^{n}+2\Delta t\mu u^{n},u^{n}\right)}_{\Gamma }+2\Delta t\epsilon ||\nabla_{\Gamma }u^{n}{\left|\right|}_{L^{2}\left(\Gamma \right)}^{2}\\ \; & =2{\left(u^{n-1}-\Delta t\beta_{\Gamma }\cdot \nabla_{\Gamma }u^{n-1}+\Delta tf^{n},u^{n}\right)}_{\Gamma }\\ \; & \leq \left|\right|u^{n-1}-\Delta t\beta_{\Gamma }\cdot \nabla_{\Gamma }u^{n-1}+\Delta tf^{n}{\left|\right|}_{L^{2}\left(\Gamma \right)}^{2}+\left|\right|u^{n}{\left|\right|}_{L^{2}\left(\Gamma \right)}^{2}\\ \; & \leq \left|\right|u^{n-1}-\Delta t\beta_{\Gamma }\cdot \nabla_{\Gamma }u^{n-1}{\left|\right|}_{L^{2}\left(\Gamma \right)}^{2}+2\Delta t{\left(u^{n-1}-\Delta t\beta_{\Gamma }\cdot \nabla_{\Gamma }u^{n-1},f^{n}\right)}_{\Gamma }+\Delta t^{2}\left|\right|f^{n}{\left|\right|}_{L^{2}\left(\Gamma \right)}^{2}+\left|\right|u^{n}{\left|\right|}_{L^{2}\left(\Gamma \right)}^{2}\\ \; & \leq \left(1+\Delta t\right)||u^{n-1}-\Delta t\beta_{\Gamma }\cdot \nabla_{\Gamma }u^{n-1}{\left|\right|}_{L^{2}\left(\Gamma \right)}^{2}+\Delta t\left(1+\Delta t\right)||f^{n}{\left|\right|}_{L^{2}\left(\Gamma \right)}^{2}+\left|\right|u^{n}{\left|\right|}_{L^{2}\left(\Gamma \right)}^{2}. \end{array}$$

For $\left|\right|f^{n}{\left|\right|}_{L^{2}\left(\Gamma \right)}^{2}$, we see that

(22) $$\begin{array}{cc} \left|\right|f^{n}{\left|\right|}_{L^{2}\left(\Gamma \right)}^{2}\; & =\int_{\Gamma }{\left(\frac{1}{\Delta t}\int_{t^{n-1}}^{t^{n}}f\left(t\right)dt\right)}^{2}dA\\ \; & =\frac{1}{\Delta t^{2}}\int_{\Gamma }\int_{t^{n-1}}^{t^{n}}\int_{t^{n-1}}^{t^{n}}f\left(t\right)f\left(s\right)dsdtdA\\ \; & \leq \frac{1}{\Delta t^{2}}\int_{\Gamma }\int_{t^{n-1}}^{t^{n}}\int_{t^{n-1}}^{t^{n}}\frac{{\left(f\left(t\right)\right)}^{2}+{\left(f\left(s\right)\right)}^{2}}{2}dsdtdA\\ \; & \leq \frac{1}{\Delta t}\int_{t^{n-1}}^{t^{n}}{\left||f\left(t\right)|\right|}_{L^{2}\left(\Gamma \right)}^{2}dt. \end{array}$$

Taking above inequality into (21), we get

(23) $$\begin{array}{cc} \; & \;\left|\right|u^{n}{\left|\right|}_{L^{2}\left(\Gamma \right)}^{2}+2\Delta t{\left(\mu u^{n},u^{n}\right)}_{\Gamma }+2\Delta t\epsilon ||\nabla_{\Gamma }u^{n}{\left|\right|}_{L^{2}\left(\Gamma \right)}^{2}\\ \; & \leq \left(1+\Delta t\right)\left(\left|\right|u^{n-1}-\Delta t\beta_{\Gamma }\cdot \nabla_{\Gamma }u^{n-1}{\left|\right|}_{L^{2}\left(\Gamma \right)}^{2}+\int_{t^{n-1}}^{t^{n}}{\left||f\left(t\right)|\right|}_{L^{2}\left(\Gamma \right)}^{2}dt\right). \end{array}$$

It follows (5) that the bound

(24) $$-2{\left(\beta_{\Gamma }\cdot \nabla_{\Gamma }u^{n},u^{n}\right)}_{\Gamma }={\left(\nabla_{\Gamma }\cdot \beta_{\Gamma },{\left(u^{n}\right)}^{2}\right)}_{\Gamma }\leq 2{\left(\mu u^{n},u^{n}\right)}_{\Gamma },$$

holds. If $\Delta t\leq \frac{2\epsilon }{\left|\right|\beta_{\Gamma }{\left|\right|}_{L^{2}\left(\Gamma \right)}^{2}}$ holds, we have

(25) $$\Delta t||\beta_{\Gamma }\cdot \nabla_{\Gamma }u^{n}{\left|\right|}_{L^{2}\left(\Gamma \right)}^{2}\leq \Delta t||\beta_{\Gamma }{\left|\right|}_{L^{2}\left(\Gamma \right)}^{2}\cdot \left|\right|\nabla_{\Gamma }u^{n}{\left|\right|}_{L^{2}\left(\Gamma \right)}^{2}\leq 2\epsilon ||\nabla_{\Gamma }u^{n}{\left|\right|}_{L^{2}\left(\Gamma \right)}^{2}.$$

Combining inequalities (24), (25) and (23), the key idea of proving Theorem 3 is obtained with

(26) $$\;\begin{array}{cc} \; & \;\left|\right|u^{n}-\Delta t\beta_{\Gamma }\cdot \nabla_{\Gamma }u^{n}{\left|\right|}_{L^{2}\left(\Gamma \right)}^{2}\\ \; & ={\left(u^{n},u^{n}\right)}_{\Gamma }-2{\left(\Delta t\beta_{\Gamma }\cdot \nabla_{\Gamma }u^{n},u^{n}\right)}_{\Gamma }+\Delta t^{2}\left|\right|\beta_{\Gamma }\cdot \nabla_{\Gamma }u^{n}{\left|\right|}_{L^{2}\left(\Gamma \right)}^{2}\\ \; & \leq {\left(u^{n},u^{n}\right)}_{\Gamma }+2\Delta t{\left(\mu u^{n},u^{n}\right)}_{\Gamma }+2\Delta t\epsilon ||\nabla_{\Gamma }u^{n}{\left|\right|}_{L^{2}\left(\Gamma \right)}^{2}\\ \; & \leq \left(1+\Delta t\right)\left(\left|\right|u^{n-1}-\Delta t\beta_{\Gamma }\cdot \nabla_{\Gamma }u^{n-1}{\left|\right|}_{L^{2}\left(\Gamma \right)}^{2}+\int_{t^{n-1}}^{t^{n}}{\left||f\left(t\right)|\right|}_{L^{2}\left(\Gamma \right)}^{2}dt\right). \end{array}$$

After repeated application of the inequality (26), we have

(27) $$\begin{array}{cc} \; & \;\left|\right|u^{n}-\Delta t\beta_{\Gamma }\cdot \nabla_{\Gamma }u^{n}{\left|\right|}_{L^{2}\left(\Gamma \right)}^{2}\\ \; & \leq {\left(1+\Delta t\right)}^{n}\left|\right|u^{0}-\Delta t\beta_{\Gamma }\cdot \nabla_{\Gamma }u^{0}{\left|\right|}_{L^{2}\left(\Gamma \right)}^{2}+\sum_{i=1}^{n}\int_{t^{i-1}}^{t^{i}}{\left(1+\Delta t\right)}^{n+1-i}{\left||f\left(t\right)|\right|}_{L^{2}\left(\Gamma \right)}^{2}dt\\ \; & \leq {\left(1+\Delta t\right)}^{n}\left(\left|\right|u^{0}-\Delta t\beta_{\Gamma }\cdot \nabla_{\Gamma }u^{0}{\left|\right|}_{L^{2}\left(\Gamma \right)}^{2}+\int_{0}^{T}{\left||f\left(t\right)|\right|}_{L^{2}\left(\Gamma \right)}^{2}dt\right). \end{array}$$

Due to ${\left(1+\Delta t\right)}^{n}\leq {\left(1+\Delta t\right)}^{N}={\left(1+\Delta t\right)}^{\frac{T}{\Delta t}}\leq e^{T}$, the following inequality obviously holds:

(28) $$\left|\right|u^{n}-\Delta t\beta_{\Gamma }\cdot \nabla_{\Gamma }u^{n}{\left|\right|}_{L^{2}\left(\Gamma \right)}^{2}\leq e^{T}\left(\left|\right|u^{0}-\Delta t\beta_{\Gamma }\cdot \nabla_{\Gamma }u^{0}{\left|\right|}_{L^{2}\left(\Gamma \right)}^{2}+\int_{0}^{T}{\left||f\left(t\right)|\right|}_{L^{2}\left(\Gamma \right)}^{2}dt\right),$$

By combining (28), (27) and (23), the proof of Theorem 3 is completed. □

### 3.3. The Surface Finite Element Method

Let ${\left\{\Gamma_{h}\right\}}_{h>0}$ be a family of discrete surfaces which is composed of plane open triangles $K_{j}$ with edge $\partial K_{j}$ and vertexes $x_{l},l=1,\ldots ,N_{h}^{v}$. The point $x_{l}$ is also the vertex points of the curved open triangle K∈Γ such that

$$\overset{N_{h}^{T}}{\underset{j=1}{\cup }}{\bar{K}}_{j}=\Gamma ,$$

and for j≠k, ${\bar{K}}_{j}\cap {\bar{K}}_{k}=\emptyset$ or common curved edge of ${\bar{K}}_{j}$ and ${\bar{K}}_{k}$ or common vertex of ${\bar{K}}_{j}$ and ${\bar{K}}_{k}$. For an interior edge $E_{j},j=1,2,\ldots ,N_{h}^{E}$, there are two triangles, $K_{l}^{j}$ and $K_{r}^{j}$, such that $\partial K_{l}^{j}\cap \partial K_{r}^{j}=E_{j}$. Following [25], we adopt the projection $P_{\Gamma_{h}}:\Gamma \to \Gamma_{h}$ which is Lipschitz continuous and $P_{\Gamma_{h}}\left(K_{j}\right)=K_{j}$ for any triangle element $K_{j}\subset \Gamma_{h}$. For any $f\in C^{0}\left(\Gamma \right)$, its projection on Γ is obtained with $f_{P_{\Gamma_{h}}}=f\circ P_{\Gamma_{h}}^{-1}$. The projection of $\beta_{P_{\Gamma_{h}}}$ on the tangent plane of the discrete surface $\Gamma_{h}$ is denoted as $\beta_{P_{\Gamma_{h}},\Gamma_{h}}$. Let finite dimensional space $S_{h}$ be a continuous function space on $\Gamma_{h}$ that is linear on each triangle $K_{j}$. Considering the variational problem (18), we obtain the MCFEM using Equation (10): For n=1,2,…,N, find $u_{h}^{n}=u_{h}\left(x,t^{n}\right)\in S_{h}$, such that

(29) $$\left\{\begin{array}{cc} {\left(\bar{\partial_{\tau }}u_{h}^{n},v_{h}\right)}_{\Gamma_{h}}+\varepsilon {\left(\nabla_{\Gamma_{h}}u_{h}^{n},\nabla_{\Gamma_{h}}v_{h}\right)}_{\Gamma_{h}}+\mu {\left(u_{h}^{n},v_{h}\right)}_{\Gamma_{h}}={\left(f_{P_{\Gamma_{h}}}^{n},v_{h}\right)}_{\Gamma_{h}} & \forall v_{h}\in S_{h},\\ u_{h}^{0}\left(x\right)=I_{h}u_{0}\left(x\right), \end{array}\right.$$

where $\bar{\partial_{\tau }}u_{h}^{n}=\frac{u_{h}^{n}-\left[u_{h}^{n-1}-\Delta t\beta_{P_{\Gamma_{h}},\Gamma_{h}}\cdot \nabla u_{h}^{n-1}\right]}{\Delta t}$, $f_{P_{\Gamma_{h}}}^{n}=\frac{1}{\Delta t}\int_{t^{n-1}}^{t^{n}}f_{P_{\Gamma_{h}}}\left(t\right)dt$, and $I_{h}$ is a piecewise linear interpolation operator.

Although method (29) is based on the idea of characteristic finite element, it utilizes Taylor’s expansion to restructure $u(\overset{ˇ}{x})$. Consequently, the MCFEM (29) degenerates into an explicit–implicit method, imposing stringent restriction on the mesh size *h* and time step Δt. Before analyzing the stability of the MCFEM (29), we need to restrict the mesh size.

**Theorem** **4.** *For any point $x_{0}\in \Gamma$, there are two triangles $K_{l}$ and $K_{r}\subset \Gamma_{h}$ such that $x_{0}\in \partial K_{l}\cap \partial K_{r}$ or $x_{0}$ is not the vertex points of $K_{l}$ and $K_{r}$. $\nu_{r}$ and $\nu_{l}$ denote the unit outward normal vectors to $K_{l}$ and $K_{r}$, respectively. If the convection velocity $\beta_{P_{\Gamma_{h},\Gamma_{h}}}\in C^{0}\left(\Gamma_{h}^{3}\right)$, then there exists a specific mesh size $h_{\kappa }$ such that the following inequality holds:*

$$|\left[\left[\nu \cdot \beta_{P_{\Gamma_{h},\Gamma_{h}}}\right]\right]\left(P_{\Gamma_{h}}\left(x_{0}\right)\right)|\leq \kappa \;\forall \;h<h_{\kappa },$$

*where $\left[\left[\nu \cdot \beta_{P_{\Gamma_{h}},\Gamma_{h}}\right]\right]\left(P_{\Gamma_{h}}\left(x_{0}\right)\right)=\lim_{h\to 0^{+}}\left[\nu_{l}\cdot \beta_{P_{\Gamma_{h},\Gamma_{h}}}{|}_{x=P_{\Gamma_{h}}\left(x_{0}\right)+h\nu_{l}}+\nu_{r}\cdot \beta_{P_{\Gamma_{h},\Gamma_{h}}}{|}_{x=P_{\Gamma_{h}}\left(x_{0}\right)+h\nu_{r}}\right]$.*

**Proof.**  By the definition, we have

(30) $$\begin{array}{cc} \; & \;\left[\left[\nu \cdot \beta_{P_{\Gamma_{h},\Gamma_{h}}}\right]\right]\left(P_{\Gamma_{h}}\left(x_{0}\right)\right)\\ \; & =\lim_{h\to 0^{+}}\left[\nu_{l}\cdot \beta_{P_{\Gamma_{h},\Gamma_{h}}}{|}_{x=P_{\Gamma_{h}}\left(x_{0}\right)+h\nu_{l}}+\nu_{r}\cdot \beta_{P_{\Gamma_{h},\Gamma_{h}}}{|}_{x=P_{\Gamma_{h}}\left(x_{0}\right)+h\nu_{r}}\right]\\ \; & =\lim_{h\to 0^{+}}\left[\nu_{l}\cdot \beta_{P_{\Gamma_{h},K_{l}}}{|}_{x=P_{\Gamma_{h}}\left(x_{0}\right)+h\nu_{l}}+\nu_{r}\cdot \beta_{P_{\Gamma_{h},K_{r}}}{|}_{x=P_{\Gamma_{h}}\left(x_{0}\right)+h\nu_{r}}\right]\\ \; & =\lim_{h\to 0^{+}}\left[\left(\nu_{l}\cdot \left(\beta_{P_{\Gamma_{h}}}-\left(\beta_{P_{\Gamma_{h}}}\cdot n_{K_{l}}\right)n_{K_{l}}\right)\right){|}_{x=P_{\Gamma_{h}}\left(x_{0}\right)+h\nu_{l}}\right]\\ & \;+\lim_{h\to 0^{+}}\left[\left(\nu_{r}\cdot \left(\beta_{P_{\Gamma_{h}}}-\left(\beta_{P_{\Gamma_{h}}}\cdot n_{K_{r}}\right)n_{K_{r}}\right)\right){|}_{x=P_{\Gamma_{h}}\left(x_{0}\right)+h\nu_{r}}\right], \end{array}$$

where $n_{K_{l}}$ and $n_{K_{r}}$ are the unit vectors normal to $K_{l}$ and $K_{r}$, respectively. Considering the orthogonality, (30) can be rewritten as

(31) $$\begin{array}{cc} \; & \;\left[\left[\nu \cdot \beta_{P_{\Gamma_{h},\Gamma_{h}}}\right]\right]\left(P_{\Gamma_{h}}\left(x_{0}\right)\right)\\ \; & =\lim_{h\to 0^{+}}\left[\nu_{l}\cdot \beta_{P_{\Gamma_{h}}}{|}_{x=P_{\Gamma_{h}}\left(x_{0}\right)+h\nu_{l}}+\nu_{r}\cdot \beta_{P_{\Gamma_{h}}}{|}_{x=P_{\Gamma_{h}}\left(x_{0}\right)+h\nu_{r}}\right]. \end{array}$$

It is obvious that

(32) $$\begin{array}{cc} \; & \;\left[\left[\nu \cdot \beta_{P_{\Gamma_{h},\Gamma_{h}}}\right]\right]\left(P_{\Gamma_{h}}\left(x_{0}\right)\right)\\ \; & =\lim_{h\to 0^{+}}\left[\nu_{l}\cdot \beta_{P_{\Gamma_{h}}}{|}_{x=P_{\Gamma_{h}}\left(x_{0}\right)+h\nu_{l}}+\nu_{r}\cdot \beta_{P_{\Gamma_{h}}}{|}_{x=P_{\Gamma_{h}}\left(x_{0}\right)+h\nu_{r}}\right]\\ \; & =\nu \cdot \beta {|}_{x=x_{0}}-\nu \cdot \beta {|}_{x=x_{0}}=0. \end{array}$$

Hence, the existence of the mesh size $h_{\kappa }$ required by Theorem 4 can be established. The proof is completed. □

Next, the stability of scheme (29) will be demonstrated.

**Theorem** **5.** *Assume that $\Delta t\leq \frac{2\epsilon }{\left|\right|\beta_{P_{\Gamma_{h},\Gamma_{h}}}{\left|\right|}_{L^{2}\left(\Gamma_{h}\right)}^{2}}$, $h\leq h_{\kappa }$ and (5) hold. Then, the solution $u_{h}^{n}$ of Problem (29) satisfies*

(33) $$\begin{array}{c} \max\left\{\left|\right|u_{h}^{n}-\Delta t\beta_{P_{\Gamma_{h},\Gamma_{h}}}\cdot \nabla_{\Gamma_{h}}u_{h}^{n}{\left|\right|}_{L^{2}\left(\Gamma_{h}\right)}^{2},\;||u_{h}^{n}{\left|\right|}_{L^{2}\left(\Gamma_{h}\right)}^{2}+2\Delta t\mu ||u_{h}^{n}{\left|\right|}_{L^{2}\left(\Gamma_{h}\right)}^{2}+2\Delta t\epsilon ||\nabla_{\Gamma_{h}}u_{h}^{n}{\left|\right|}_{L^{2}\left(\Gamma_{h}\right)}^{2}\right\}\\ \leq e^{T}\left(\left|\right|u_{h}^{0}-\Delta t\beta_{P_{\Gamma_{h},\Gamma_{h}}}\cdot \nabla_{\Gamma_{h}}u_{h}^{0}{\left|\right|}_{L^{2}\left(\Gamma_{h}\right)}^{2}+\int_{0}^{T}\left|\right|f_{P_{\Gamma_{h}}}\left(t\right){\left|\right|}_{L^{2}\left(\Gamma_{h}\right)}^{2}dt\right), \end{array}$$

*for n=1,2,…,N.*

**Proof.**  Choose $v_{h}=2\Delta tu_{h}^{n}$ in (29) so that

(34) $$\begin{array}{cc} \; & \;{\left(2u_{h}^{n}+2\Delta t\cdot \mu u_{h}^{n},u_{h}^{n}\right)}_{\Gamma_{h}}+2\Delta t\epsilon ||\nabla_{\Gamma_{h}}u_{h}^{n}{\left|\right|}_{L^{2}\left(\Gamma_{h}\right)}^{2}\\ \; & =2{\left(u_{h}^{n-1}-\Delta t\beta_{P_{\Gamma_{h},\Gamma_{h}}}\cdot \nabla_{\Gamma_{h}}u_{h}^{n-1}+\Delta tf_{P_{\Gamma_{h}}}^{n},u_{h}^{n}\right)}_{\Gamma_{h}}\\ \; & \leq \left|\right|u_{h}^{n-1}-\Delta t\beta_{P_{\Gamma_{h},\Gamma_{h}}}\cdot \nabla_{\Gamma_{h}}u_{h}^{n-1}+\Delta tf_{P_{\Gamma_{h}}}^{n}{\left|\right|}_{L^{2}\left(\Gamma_{h}\right)}^{2}+\left|\right|u_{h}^{n}{\left|\right|}_{L^{2}\left(\Gamma_{h}\right)}^{2}. \end{array}$$

Similarly to Theorem 3, we have

(35) $$\begin{array}{cc} \; & \;\left|\right|u_{h}^{n}-\Delta t\beta_{P_{\Gamma_{h},\Gamma_{h}}}\cdot \nabla_{\Gamma_{h}}u_{h}^{n}{\left|\right|}_{L^{2}\left(\Gamma_{h}\right)}^{2}+2\Delta t{\left(\mu u_{h}^{n}+\beta_{P_{\Gamma_{h},\Gamma_{h}}}\cdot \nabla_{\Gamma_{h}}u_{h}^{n},u_{h}^{n}\right)}_{\Gamma_{h}}\\ & \leq \left(1+\Delta t\right)\left(\left|\right|u_{h}^{n-1}-\Delta t\beta_{P_{\Gamma_{h},\Gamma_{h}}}\cdot \nabla_{\Gamma_{h}}u_{h}^{n-1}{\left|\right|}_{L^{2}\left(\Gamma_{h}\right)}^{2}+\int_{t^{n-1}}^{t^{n}}\left|\right|f_{P_{\Gamma_{h}}}\left(t\right){\left|\right|}_{L^{2}\left(\Gamma_{h}\right)}^{2}dt\right). \end{array}$$

We claim that ${\left(\mu u_{h}^{n}+\beta_{P_{\Gamma_{h},\Gamma_{h}}}\cdot \nabla_{\Gamma_{h}}u_{h}^{n},u_{h}^{n}\right)}_{\Gamma_{h}}\geq 0$. Owing to (5), the following inequality holds:

(36) $$\;\;\;\begin{array}{cc} \; & \;2{\left(\mu u_{h}^{n}+\beta_{P_{\Gamma_{h},\Gamma_{h}}}\cdot \nabla_{\Gamma_{h}}u_{h}^{n},u_{h}^{n}\right)}_{\Gamma_{h}}\\ \; & \geq 2\kappa {\left(u_{h}^{n},u_{h}^{n}\right)}_{\Gamma_{h}}+{\left(\nabla_{\Gamma_{h}}\cdot \beta_{P_{\Gamma_{h},\Gamma_{h}}},{\left(u_{h}^{n}\right)}^{2}\right)}_{\Gamma_{h}}+2{\left(\beta_{P_{\Gamma_{h},\Gamma_{h}}}\cdot \nabla_{\Gamma_{h}}u_{h}^{n},u_{h}^{n}\right)}_{\Gamma_{h}}. \end{array}$$

Since the discrete surface $\Gamma_{h}$ is a piecewise linear approximation of Γ, the unit outward normal vectors to any adjacent triangles, $K_{l}^{j}$ and $K_{r}^{j}$, are discontinuous at a common edge $E_{j}$. Consequently, the last two terms on the right side of inequality (36) are not equal to 0. Using Theorem 1, we obtain

(37) $$\begin{array}{cc} \; & \;{\left(\nabla_{\Gamma_{h}}\cdot \beta_{P_{\Gamma_{h},\Gamma_{h}}},{\left(u_{h}^{n}\right)}^{2}\right)}_{\Gamma_{h}}+2{\left(\beta_{P_{\Gamma_{h},\Gamma_{h}}}\cdot \nabla_{\Gamma_{h}}u_{h}^{n},u_{h}^{n}\right)}_{\Gamma_{h}}\\ \; & =\int_{\Gamma_{h}}\nabla_{\Gamma_{h}}\left(\beta_{P_{\Gamma_{h},\Gamma_{h}}}{\left(u_{h}^{n}\right)}^{2}\right)dA_{h}\\ \; & =\sum_{j=1}^{N_{h}^{T}}\int_{K_{j}}\nabla_{K_{j}}\left(\beta_{P_{\Gamma_{h},K_{j}}}{\left(u_{h}^{n}\right)}^{2}\right)dA_{h}\\ \; & =\sum_{j=1}^{N_{h}^{T}}\left[\int_{K_{j}}H_{K_{j}}{\left(u_{h}^{n}\right)}^{2}\beta_{P_{\Gamma_{h},K_{j}}}\cdot n_{K_{j}}dA_{h}+\int_{\partial K_{j}}{\left(u_{h}^{n}\right)}^{2}\beta_{P_{\Gamma_{h},K_{j}}}\cdot \nu_{\partial K_{j}}d\sigma_{h}\right], \end{array}$$

where $n_{K_{j}}$ is the unit vector normal to $K_{j}$, and $\nu_{\partial K_{j}}$ is the unit normal vector of boundary $\partial K_{j}$ and tangent to $K_{j}$. Note that $dA_{h}$ and $d\sigma_{h}$ are the measures of $\Gamma_{h}$ and interior edge $\partial K_{j}$, respectively. The mean curvature of triangular element $K_{j}$ is defined as $H_{K_{j}}$. It follows that $K_{j}$ is a triangular plane and that $H_{K_{j}}$ is equal to 0. By choosing the mesh size $h<h_{\kappa }$ and applying Theorem 4, a lower bound for the inequality (37) can be obtained with

(38) $$\begin{array}{cc} \; & \;{\left(\nabla_{\Gamma_{h}}\cdot \beta_{P_{\Gamma_{h},\Gamma_{h}}},{\left(u_{h}^{n}\right)}^{2}\right)}_{\Gamma_{h}}+2{\left(\beta_{P_{\Gamma_{h},\Gamma_{h}}}\cdot \nabla_{\Gamma_{h}}u_{h}^{n},u_{h}^{n}\right)}_{\Gamma_{h}}\\ \; & =\sum_{j=1}^{N_{h}^{T}}\int_{\partial K_{j}}{\left(u_{h}^{n}\right)}^{2}\beta_{P_{\Gamma_{h},K_{j}}}\cdot \nu_{\partial K_{j}}d\sigma_{h}\\ \; & =\sum_{j=1}^{N_{h}^{E}}\int_{\partial K_{l}^{j}\cap \partial K_{r}^{j}}{\left(u_{h}^{n}\right)}^{2}\left[\beta_{P_{\Gamma_{h},K_{l}^{j}}}\cdot \nu_{\partial K_{l}^{j}}+\beta_{P_{\Gamma_{h},K_{r}^{j}}}\cdot \nu_{\partial K_{r}^{j}}\right]d\sigma_{h}\\ \; & \geq -\kappa \sum_{j=1}^{N_{h}^{E}}\int_{\partial K_{l}^{j}\cap \partial K_{r}^{j}}{\left(u_{h}^{n}\right)}^{2}d\sigma_{h}=-\kappa \sum_{j=1}^{N_{h}^{T}}\int_{\partial K_{j}}{\left(u_{h}^{n}\right)}^{2}d\sigma_{h}. \end{array}$$

If we plug inequality (38) into (36), we obtain

(39) $$2{\left(\mu u_{h}^{n}+\beta_{P_{\Gamma_{h},\Gamma_{h}}}\cdot \nabla_{\Gamma_{h}}u_{h}^{n},u_{h}^{n}\right)}_{\Gamma_{h}}\geq 2\kappa \sum_{j=1}^{N_{h}^{T}}\int_{\partial K_{j}}{\left(u_{h}^{n}\right)}^{2}d\sigma_{h}-\kappa \sum_{j=1}^{N_{h}^{T}}\int_{\partial K_{j}}{\left(u_{h}^{n}\right)}^{2}d\sigma_{h}\geq 0.$$

Others are similar to Theorem 3. The proof of Theorem 5 is completed. □

### 3.4. The Analysis of Reconstruction Methods in MCFEM and CFEM

The reconstruction method proposed in Section 3.1 is different from that employed in the CFEM [29], as the scheme of the MCFEM does not involve projection. This method pre-processes the convection velocity β to guarantee that the backtracking characteristic point $\overset{ˇ}{x}$ is situated on the tangent plane $T_{x}$ of the surface Γ. Taylor expansion is utilized in the MCFEM to maintain a second-order spatial accuracy. However, it also transforms the MCFEM into an explicit–implicit method, reducing its stability compared to the CFEM.

As described in Section 3.1, the reconstruction method employed by the CFEM involves projecting the backtracking characteristic point $\overset{ˇ}{x}$ and its closer mesh points onto tangent planes $T_{c}$, thereby generating a novel local mesh. This also makes the CFEM more stable than the MCFEM. Since the characteristic finite element employs the definition domain of the solution *u* to be a neighborhood U(Γ,δ) containing the surface Γ, the value of *u* should be different at the same normal vector on Γ. This is contrary to the more widely accepted understanding in the use of conventional surface finite element method described in [25].

Employing the notations given in Section 3.1, the errors in the reconstruction method of the CFEM consist of $|u\left(\overset{ˇ}{x}\right)-u\left(x^{*}\right)|$ and $|u\left(x^{*}\right)-u_{h}\left(x^{*}\right)|$. Obviously,

(40) $$|u\left(\overset{ˇ}{x}\right)-u\left(x^{*}\right)|\leq \max_{x\in U(\Gamma ,\delta )}|\nabla u\left(x\right)\left|\cdot \right|\overset{ˇ}{x}-x^{*}|.$$

Here, $|\overset{ˇ}{x}-x^{*}|$ is not easy to estimate. The only certainty is that $|\overset{ˇ}{x}-x^{*}|$ is related to the time step Δt. Similarly, the error of $|u\left(x^{*}\right)-u_{h}\left(x^{*}\right)|$ encompasses errors arising from the projection of mesh points onto the tangent plane $T_{c}$, in addition to interpolation errors. Consequently, the presence of $|\overset{ˇ}{x}-x^{*}|$ and the projection error in $|u\left(x^{*}\right)-u_{h}\left(x^{*}\right)|$ results in a deterioration in the convergence order of the CFEM when compared to the MCFEM.

## 4. Numerical Examples

In this section, we evaluate the precision of the MCFEM under both diffusion-dominated and convection-dominated conditions. Then, the impact of variation in curvature and multi-connected surfaces on the MCFEM is verified. To simulate the phenomenon of pollutant injection on torus, a discontinuous source term problem is employed. Furthermore, the nonlinear convection velocity’s impact on the MCFEM is evaluated by applying the Burgers equation on a peanut-shaped surface. Finally, the impact of random initial conditions on our method is confirmed by employing the convection Allen–Cahn equation on other multi-connected surface. The $L^{2}$ errors (denoted by ${\mathrm{Err}}_{L^{2}}=||u\left(T\right)-u_{h}^{N}{\left|\right|}_{L^{2}\left(\Gamma_{h}\right)}$) and the $H^{1}$ errors (denoted by ${\mathrm{Err}}_{H^{1}}=||u\left(T\right)-u_{h}^{N}{\left|\right|}_{H^{1}\left(\Gamma_{h}\right)}$) of the numerical solutions are computed, respectively.

### 4.1. Accuracy Test on the Sphere

Initially, we will evaluate the spatial accuracy of the MCFEM and compare it with the CFEM, assuming a time step of $\Delta t\approx h^{2}$. Consider the CRD Equation (4) on a sphere

(41) $$\psi \left(x,y,z\right)=x^{2}+y^{2}+z^{2}-0.25=0,$$

where the reaction coefficient μ is set to 1 and the convection velocity β is set to [0,0,0.5]. The exact solution can be expressed as follows:

(42) $$u\left(x,y,z,t\right)=t^{2}\left(1-\tanh\left(\frac{z}{\sqrt{\epsilon }}\right)\right).$$

When the diffusion parameter $\epsilon \ll {\left||\beta |\right|}_{L^{2}\left(\Gamma \right)}$, the exact solution *u* is discontinuous at the equator of sphere (42), resulting in a convection-dominated (singular perturbation) problem. To investigate this phenomenon, we set the diffusion parameter ϵ to 1, 1 $\times \;10^{-1}$, 1 $\times \;10^{-2}$, 1 $\times \;10^{-3}$ and 1 $\times \;10^{-4}$, respectively. The corresponding results are presented in Table 1, Table 2, Table 3, Table 4 and Table 5.

The $L^{2}$ errors and $H^{1}$ errors of the MCFEM in Table 1 show the same trend as that of the CFEM. When h>2.67 $\times \;10^{-2}$, the mesh size *h* is not fine enough to ignore the existence of geometric errors. Consequently, the outcomes for h≥2.67 $\times \;10^{-2}$ deviate from the anticipated results. As the mesh size *h* diminishes, the $L^{2}$ errors convergence order of the MCFEM attains 2, while the $H^{1}$ errors convergence order attains 1. This observation indicates that the MCFEM has second-order spatial accuracy when diffusion is dominant.

With the decrease in parameter ϵ, the diffusion of the equation begins to weaken and convection gradually dominates. The $L^{2}$ errors and $H^{1}$ errors of the MCFEM, as shown in Table 2 and Table 3, are smaller than those of the CFEM. When ϵ=1 $\times \;10^{-3}$, the discontinuity of the exact solution *u* is obviously enhanced, resulting in a noticeable increase in the error of the MCFEM compared to ϵ>1 $\times \;10^{-3}$. Fortunately, the MCFEM still maintains second-order spatial accuracy in this case. Compared with the MCFEM, the $L^{2}$ errors convergence order of the CFEM fails to reach 2 when ϵ=1 $\times \;10^{-1}$. This decrease occurs as a result of the discrepancy in *u* on the same normal vector when it is convection-dominated. The projection error in the CFEM’s reconstruction method cannot be disregarded. However, the MCFEM only involves Taylor expansion and surface finite element discretization without introducing other spatial errors. This is why our method ensures that the $L^{2}$ errors convergence order is $O(h^{2})$.

The continuity of the exact solution *u* is significantly compromised when the diffusion parameter ϵ=1 $\times \;10^{-4}$ as opposed to ϵ=1 $\times \;10^{-3}$. Consequently, the numerical solutions of the MCFEM and the CFEM under coarse mesh have obvious oscillation, as shown in Table 5. Analogous to the CFEM, the MCFEM demonstrates stability solely when h=1.32 $\times \;10^{-2}$. This observation underscores the influence of continuity on the mesh requirements for the MCFEM. Thus, it becomes imperative to employ a finer mesh size *h* to ensure the efficacy of the MCFEM when the diffusion parameter ϵ is very small.

**Figure 3:**
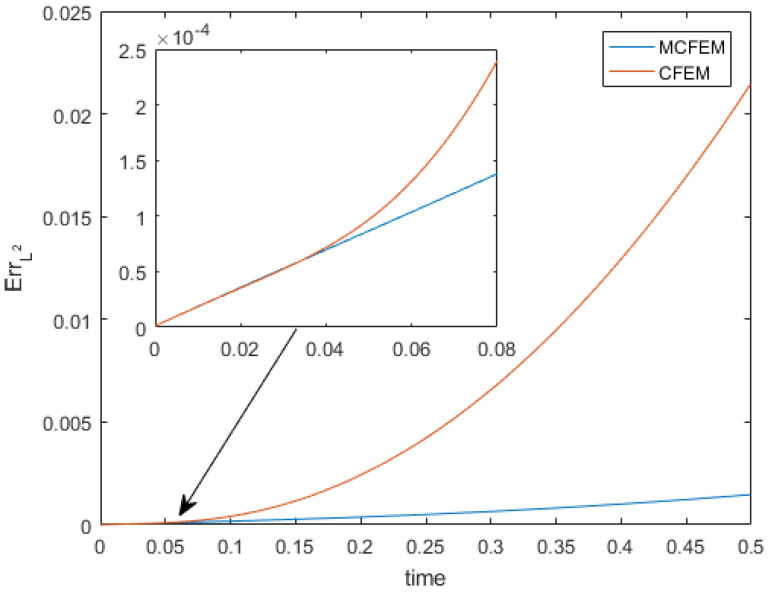
The $L^{2}$ errors of various methods with time at ϵ=1 $\times \;10^{-3}$.

**Figure 4:**
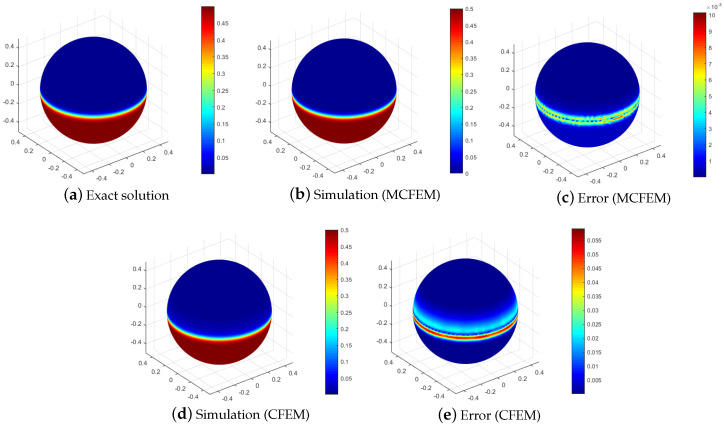
The simulations and corresponding errors of various methods with ϵ=1 $\times \;10^{-3}$ at T=0.5.

To visually reveal the distinctions between our proposed MCFEM and CFEM, we present a comparison of numerical solutions and error for various methods at h=2.67 $\times \;10^{-2}$, as illustrated in Figure 3 and Figure 4. As depicted in Figure 3, the $L^{2}$ errors of both the MCFEM and CFEM exhibit an upward trend over time. Prior to t=0.04, the $L^{2}$ errors of the MCFEM is equivalent to that of the CFEM. With the increase in time, the $L^{2}$ errors growth rate of the CFEM is obviously faster than that of the MCFEM. This observation indicates that the accumulation of errors over time is significantly smoother for the MCFEM compared to the CFEM. The errors at the final moment of the MCFEM and the CFEM are shown in the subgraph (c,e) in Figure 4. We can see that the error distribution of the MCFEM is sparsely concentrated near the equator and is an order of magnitude smaller than that of the CFEM. In contrast, the CFEM produces a narrow error band near the equator with slight oscillations above it. These findings suggest that the MCFEM produces less error than the CFEM once it reaches stability.

Furthermore, we also considered the curvature variation surfaces,

(43) $$\begin{array}{c} \mathrm{tooth}:\psi \left(x,y,z\right)=x^{4}+y^{4}+z^{4}-\left(x^{2}+y^{2}+z^{2}\right)=0, \end{array}$$

and multi-connected surface,

(44) $$\begin{array}{c} \mathrm{torus}:\psi \left(x,y,z\right)={\left(0.5-\sqrt{x^{2}+y^{2}}\right)}^{2}+z^{2}-0.1^{2}=0. \end{array}$$

**Figure 5:**
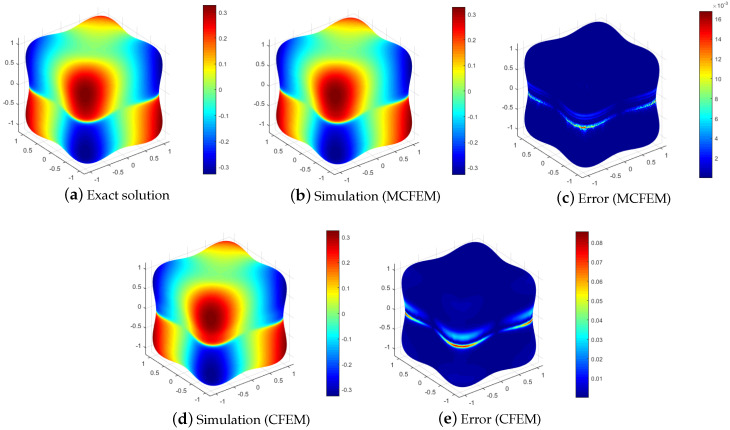
The simulations and corresponding errors of various methods with ϵ=1 $\times \;10^{-3}$ and h=3.13 $\times \;10^{-2}$ on a tooth.

**Figure 6:**
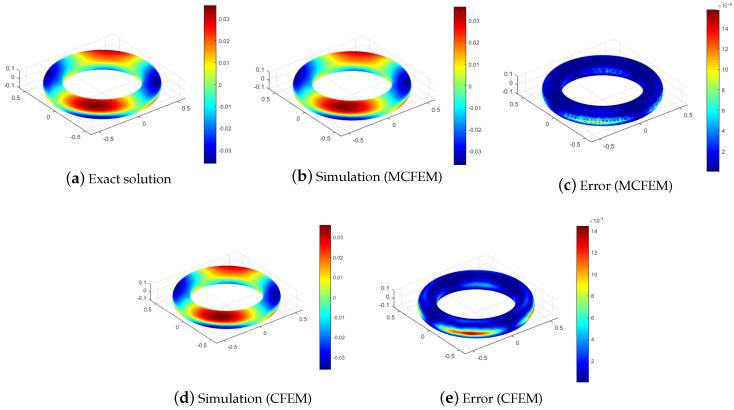
The simulations and corresponding errors of various methods with ϵ=1 $\times \;10^{-3}$ and h=2.5 $\times \;10^{-2}$ on a torus.

The exact solution will be modified with

(45) $$u\left(x,y,z,t\right)=e^{-t}\frac{xy}{\pi }\arctan\left(\frac{z}{\sqrt{\epsilon }}\right),$$

while maintaining the convection velocity β and keeping the reaction coefficient μ unchanged. The diffusion parameter ϵ is set to 1 $\times \;10^{-3}$, and the time step Δt is set to $2h^{2}$. Additionally, the exact solution of *u* at T=0.5 is simulated using the MCFEM and the CFEM on a tooth and a torus, respectively. The corresponding results are shown in Figure 5 and Figure 6.

As depicted in Figure 5 and Figure 6, the error is centered at z=0, aligning with the anticipated results. Our proposed MCFEM demonstrates its efficacy in handling surfaces with varying curvatures and multi-connected topologies. The errors of both the MCFEM and the CFEM suggest that the stabilized MCFEM outperforms the CFEM in terms of accuracy.

### 4.2. The Discontinuous Source Term Problem on Torus

Here, the MCFEM will be utilized to simulate the movement of pollutants on a torus, which is tantamount to resolving the convection-dominated problem that contains discontinuous source terms. The level set function expression of the torus is indicated in Equation (44). In order to maintain stability within the MCFEM, it is imperative to restrict the mesh size to h=1.25 $\times \;10^{-2}$ and the time step to Δt=1 $\times \;10^{-3}$. Additionally, the reaction coefficient should be fixed at μ=1, the parameter at ϵ=1 $\times \;10^{-3}$, and the convection velocity at β=[−y,x,0]. Furthermore, the pollutant is yet to be introduced into the torus at time t=0; therefore, an initial value of $u_{0}=0$ is selected. Four points ${\left\{x_{s}\right\}}_{s=1}^{4}$ on the torus are arbitrarily selected, and the pollutants are continuously injected at these points ${\left\{x_{s}\right\}}_{s=1}^{4}$, respectively, to create a discontinuous source term:

(46) $$f=\left\{\begin{array}{cc} 5, & |x-x_{s}|<0.03,\;s=1,2,3,4,\;x\in \Gamma ,\\ 0, & \mathrm{otherwise}. \end{array}\right.$$

**Figure 7:**
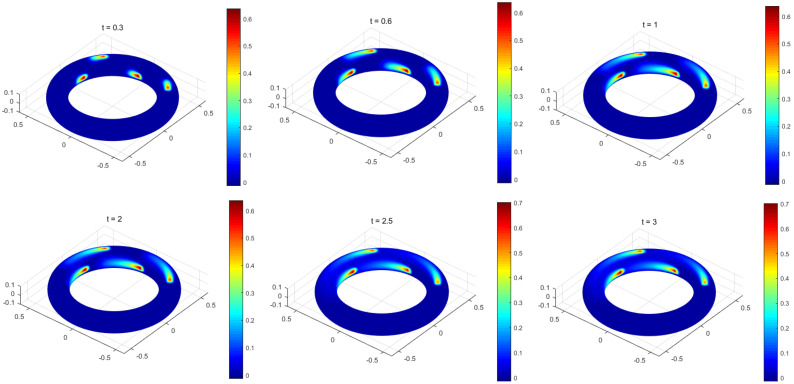
The simulations of discontinuous source term problem at various time in Example Section 4.2.

According to the findings presented in Figure 7, pollutants form four stable regions on the torus are under the influence of discontinuous source terms and convection velocity. This observation illustrates that the numerical solutions acquired through the MCFEM demonstrate comparable physical phenomena with the ones obtained from the CFEM in [29].

### 4.3. The Burgers Equation on Peanut-Shaped Surface

To investigate the impact of nonlinear problems on our methods, we selected the convection velocity β=[u,0,0]. Assigning the diffusion parameter ϵ=1 $\times \;10^{-3}$, reaction coefficient μ=0, and setting the source term to f=0, the problem transforms into a typical Burgers problem on surfaces. Without loss of generality, a peanut-shaped surface

(47) $$\psi \left(x,y,z\right)=\left({\left(2x-1\right)}^{2}+4y^{2}+4z^{2}\right)\left({\left(2x+1\right)}^{2}+4y^{2}+4z^{2}\right)-1.5=0,$$

is selected and the initial condition is as follows:

(48) $$u_{0}=\left\{\begin{array}{cc} -\sin(2\pi x), & |y|\leq 0.5,\\ 0, & \mathrm{otherwise}. \end{array}\right.$$

The corresponding mesh size h=1 $\times \;10^{-2}$ and time step Δt=1 $\times \;10^{-3}$. Since the convection velocity β depends on time, the velocity β at the current moment is approximated by the velocity β at the previous moment.

**Figure 8:**
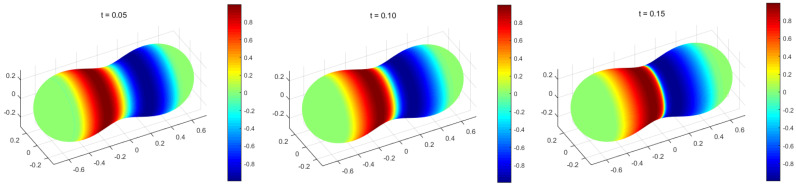
The MCFEM for simulating Burgers problem in Section 4.3.

**Figure 9:**
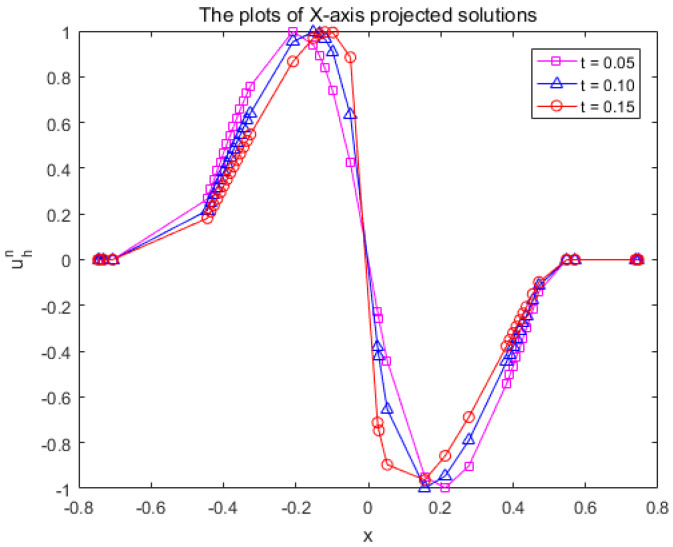
The X-axis projection of the numerical solution $u_{h}^{n}$ is restricted to $|y|<4\;\times \;10^{-4}$ in Section 4.3.

The findings are presented in Figure 8. As time progresses, the numerical solution displays a marked modification at the centre of the peanut. To depict this trend visually, we projected the numerical solution $u_{h}^{n}$, where $|y|<4\;\times \;10^{-4}$, onto the X-axis, and the result is illustrated in Figure 9. The wave clearly propagates forward over time, and the gradient near x=0 exhibits progressive increments. The results of the calculations bear similarity to the one-dimensional case [32], implying the applicability of the MCFEM in addressing nonlinear convection-dominated problem on surfaces.

### 4.4. The Convection Allen–Cahn Equation on Multi-Connected Surface

In this example, we will analyze the impact of random initial conditions on the MCFEM using the convection Allen–Cahn equation,

(49) $$\partial_{\tau }u-\varepsilon \Delta_{\Gamma }u=f\left(u\right),$$

on a multi-connected surface,

(50) $$\psi \left(x,y,z\right)={\left(x^{2}+y^{2}-4\right)}^{2}+{\left(x^{2}+z^{2}-4\right)}^{2}+{\left(z^{2}+y^{2}-4\right)}^{2}+{\left(x^{2}-1\right)}^{2}+{\left(y^{2}-1\right)}^{2}+{\left(z^{2}-1\right)}^{2}-15=0.$$

**Figure 10:**
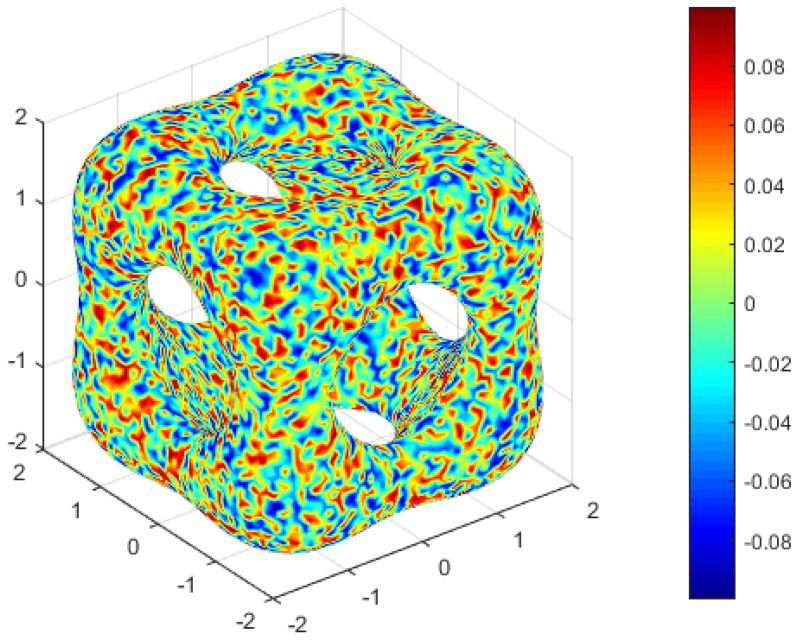
The initial condition of convection Allen–Cahn equation in Section 4.4.

The convection velocity β=[0,0,−2], the diffusion parameter ε=1 $\times \;10^{-3}$ and nonlinear function $f\left(u\right)=\frac{1}{\varepsilon }\left(u^{3}-u\right)$ are selected. The initial condition is randomly chosen within the range of −0.1 to 0.1, as depicted in Figure 10. We define the discrete free energy as

(51) $$E_{h}^{n}=\int_{\Gamma_{h}}\varepsilon {\left|\nabla_{\Gamma_{h}}u_{h}^{n}\right|}^{2}+F\left(u_{h}^{n}\right)d\sigma ,$$

where $F\left(u_{h}^{n}\right)=\frac{1}{\varepsilon }{\left({\left(u_{h}^{n}\right)}^{2}-1\right)}^{2}$. Additionally, the nonlinear function $f(u_{h}^{n})$ can be approximated as $f\left(u_{h}^{n-1}\right)+\frac{2}{\varepsilon }\left(u_{h}^{n}-u_{h}^{n-1}\right)$.

**Figure 11:**
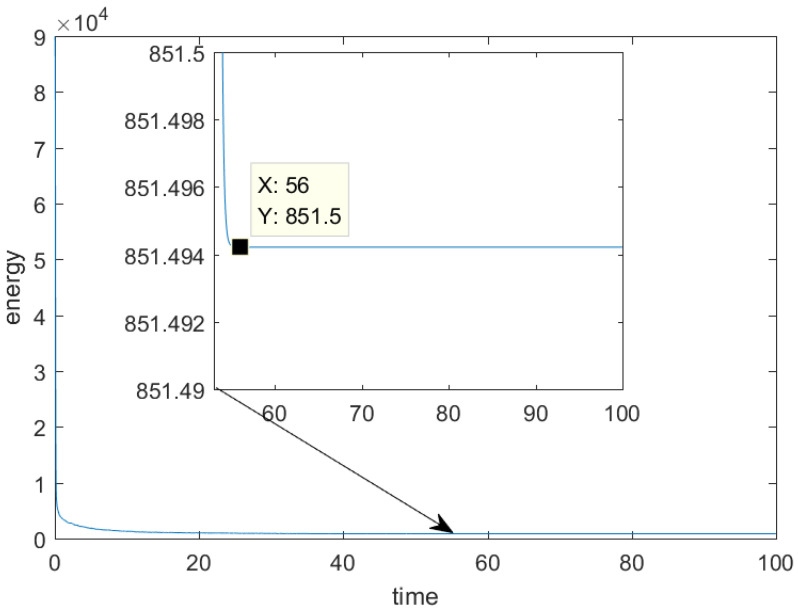
The evolution of energy of convection Allen–Cahn equation with time in Section 4.4.

**Figure 12:**
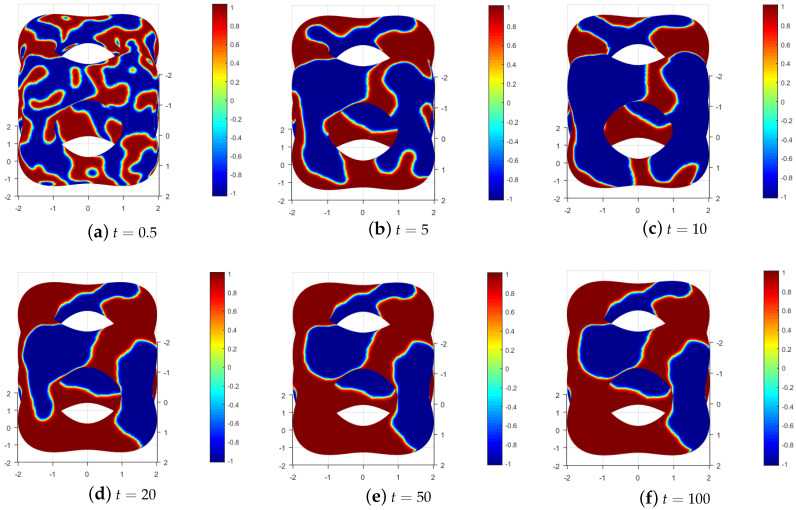
Time snapshot of the numerical solution for convection Allen–Cahn equation in Section 4.4.

The decrease in energy is a well-established property of the convection Allen–Cahn equation. To observe the energy variation in the numerical solution, we control the mesh size h=1.08 $\times \;10^{-1}$ and the time step Δt=1 $\times \;10^{-2}$ to obtain Figure 11. The presented data in Figure 11 demonstrate a decrease in discrete energy over time, ultimately reaching a state of stability at t=56. This observation indicates that the random initial condition does not significantly impact the effectiveness of the MCFEM. To visually depict the progression from the initial condition to the steady state, numerical solutions at various time points within the range of [0,100] were extracted and uniformly rotated. The outcomes are illustrated in Figure 12, revealing that the time trend of phase separation is consistent with the results observed in Figure 11.

## 5. Conclusions

This paper introduces a modified characteristic finite element method that exhibits second-order spatial accuracy. Our method employs Taylor expansion to reconstruct the solution beyond the surface in the characteristic direction. In contrast, the CFEM’s [29] reconstruction method introduces additional spatial errors, resulting in a lower spatial convergence order compared to our method. The reason for this phenomenon is that the definition domain of the solution has been extended from the surface to the neighborhood containing the surface with the characteristic finite element method. Consequently, the solutions along the same normal vector remain unequal by default, which is contrary to [25]. Despite the superior spatial accuracy of our proposed MCFEM in comparison to the CFEM, this advantage comes at the expense of stability. The reason for this sacrifice in stability is that our reconstruction method transforms the characteristic finite element method to an explicit–implicit method.

## Figures and Tables

**Table 1:** The errors of different methods with ϵ=1 and $\frac{2\epsilon }{{\left||\beta |\right|}_{L^{2}\left(\Gamma \right)}^{2}}=1.27$.

*h*	MCFEM				CFEM			
	${\mathrm{Err}}_{L^{2}}$	Rate	${\mathrm{Err}}_{H^{1}}$	Rate	${\mathrm{Err}}_{L^{2}}$	Rate	${\mathrm{Err}}_{H^{1}}$	Rate
2.04 $\times \;10^{-1}$	2.42 $\times \;10^{-2}$	–	4.33 $\times \;10^{-2}$	–	2.30 $\times \;10^{-2}$	–	4.23 $\times \;10^{-2}$	–
1.08 $\times \;10^{-1}$	1.44 $\times \;10^{-2}$	0.82	2.70 $\times \;10^{-2}$	0.74	1.49 $\times \;10^{-2}$	0.69	2.80 $\times \;10^{-2}$	0.65
5.38 $\times \;10^{-2}$	3.63 $\times \;10^{-3}$	1.99	1.05 $\times \;10^{-2}$	1.37	3.85 $\times \;10^{-3}$	1.96	1.08 $\times \;10^{-2}$	1.37
2.67 $\times \;10^{-2}$	4.27 $\times \;10^{-4}$	3.05	4.57 $\times \;10^{-3}$	1.18	3.98 $\times \;10^{-4}$	3.23	4.57 $\times \;10^{-3}$	1.23
1.32 $\times \;10^{-2}$	1.05 $\times \;10^{-4}$	2.00	2.25 $\times \;10^{-3}$	1.01	1.09 $\times \;10^{-4}$	1.85	2.26 $\times \;10^{-3}$	1.00

**Table 2:** The errors of different methods with ϵ=1 $\times \;10^{-1}$ and $\frac{2\epsilon }{{\left||\beta |\right|}_{L^{2}\left(\Gamma \right)}^{2}}=1.27$ $\times \;10^{-1}$.

*h*	MCFEM				CFEM			
	${\mathrm{Err}}_{L^{2}}$	Rate	${\mathrm{Err}}_{H^{1}}$	Rate	${\mathrm{Err}}_{L^{2}}$	Rate	${\mathrm{Err}}_{H^{1}}$	Rate
2.04 $\times \;10^{-1}$	2.65 $\times \;10^{-2}$	–	1.34 $\times \;10^{-1}$	–	2.19 $\times \;10^{-2}$	–	1.31 $\times \;10^{-1}$	–
1.08 $\times \;10^{-1}$	1.68 $\times \;10^{-2}$	0.71	6.86 $\times \;10^{-2}$	1.05	2.01 $\times \;10^{-2}$	0.14	7.76 $\times \;10^{-2}$	0.82
5.38 $\times \;10^{-2}$	4.24 $\times \;10^{-3}$	1.99	3.19 $\times \;10^{-2}$	1.11	6.01 $\times \;10^{-3}$	1.74	3.58 $\times \;10^{-2}$	1.12
2.67 $\times \;10^{-2}$	4.75 $\times \;10^{-4}$	3.12	1.54 $\times \;10^{-2}$	1.04	1.04 $\times \;10^{-3}$	2.51	1.63 $\times \;10^{-2}$	1.12
1.32 $\times \;10^{-2}$	1.17 $\times \;10^{-4}$	2.00	7.59 $\times \;10^{-3}$	1.01	5.55 $\times \;10^{-4}$	0.89	8.15 $\times \;10^{-3}$	0.99

**Table 3:** The errors of different methods with ϵ=1 $\times \;10^{-2}$ and $\frac{2\epsilon }{{\left||\beta |\right|}_{L^{2}\left(\Gamma \right)}^{2}}=1.27$ $\times \;10^{-2}$.

*h*	MCFEM				CFEM			
	${\mathrm{Err}}_{L^{2}}$	Rate	${\mathrm{Err}}_{H^{1}}$	Rate	${\mathrm{Err}}_{L^{2}}$	Rate	${\mathrm{Err}}_{H^{1}}$	Rate
2.04 $\times \;10^{-1}$	4.15 $\times \;10^{-2}$	–	7.20 $\times \;10^{-1}$	–	4.77 $\times \;10^{-2}$	–	7.31 $\times \;10^{-1}$	–
1.08 $\times \;10^{-1}$	1.90 $\times \;10^{-2}$	1.22	3.39 $\times \;10^{-1}$	1.18	3.48 $\times \;10^{-2}$	0.49	4.25 $\times \;10^{-1}$	0.85
5.38 $\times \;10^{-2}$	4.73 $\times \;10^{-3}$	2.00	1.64 $\times \;10^{-1}$	1.05	1.54 $\times \;10^{-2}$	1.18	2.29 $\times \;10^{-1}$	0.89
2.67 $\times \;10^{-2}$	6.64 $\times \;10^{-4}$	2.80	8.04 $\times \;10^{-2}$	1.01	6.59 $\times \;10^{-3}$	1.21	1.17 $\times \;10^{-1}$	0.96
1.32 $\times \;10^{-2}$	1.63 $\times \;10^{-4}$	2.00	3.94 $\times \;10^{-2}$	1.02	3.43 $\times \;10^{-3}$	0.93	5.99 $\times \;10^{-2}$	0.95

**Table 4:** The errors of different methods with ϵ=1 $\times \;10^{-3}$ and $\frac{2\epsilon }{{\left||\beta |\right|}_{L^{2}\left(\Gamma \right)}^{2}}=1.27$ $\times \;10^{-3}$.

*h*	MCFEM				CFEM			
	${\mathrm{Err}}_{L^{2}}$	Rate	${\mathrm{Err}}_{H^{1}}$	Rate	${\mathrm{Err}}_{L^{2}}$	Rate	${\mathrm{Err}}_{H^{1}}$	Rate
2.04 $\times \;10^{-1}$	1.03 $\times \;10^{-1}$	–	2.86 $\times \;10^{0}$	–	1.09 $\times \;10^{-1}$	–	2.65 $\times \;10^{0}$	–
1.08 $\times \;10^{-1}$	4.95 $\times \;10^{-2}$	1.15	2.40 $\times \;10^{0}$	0.27	7.23 $\times \;10^{-2}$	0.65	2.15 $\times \;10^{0}$	0.33
5.38 $\times \;10^{-2}$	1.18 $\times \;10^{-2}$	2.07	1.32 $\times \;10^{0}$	0.92	4.10 $\times \;10^{-2}$	0.82	1.55 $\times \;10^{0}$	0.47
2.67 $\times \;10^{-2}$	1.47 $\times \;10^{-3}$	2.97	4.54 $\times \;10^{-1}$	1.50	2.14 $\times \;10^{-2}$	0.92	8.29 $\times \;10^{-1}$	0.89
1.32 $\times \;10^{-2}$	3.37 $\times \;10^{-4}$	2.10	2.21 $\times \;10^{-1}$	1.03	1.12 $\times \;10^{-2}$	0.93	4.31 $\times \;10^{-1}$	0.93

**Table 5:** The errors of different methods with ϵ=1 $\times \;10^{-4}$ and $\frac{2\epsilon }{{\left||\beta |\right|}_{L^{2}\left(\Gamma \right)}^{2}}=1.27$ $\times \;10^{-4}$.

*h*	MCFEM				CFEM			
	${\mathrm{Err}}_{L^{2}}$	Rate	${\mathrm{Err}}_{H^{1}}$	Rate	${\mathrm{Err}}_{L^{2}}$	Rate	${\mathrm{Err}}_{H^{1}}$	Rate
2.04 $\times \;10^{-1}$	1.04 $\times \;10^{-1}$	–	5.90	–	1.54 $\times \;10^{-1}$	–	5.33	–
1.08 $\times \;10^{-1}$	1.53 $\times \;10^{-1}$	−0.61	5.49	0.11	1.07 $\times \;10^{-1}$	0.56	5.06	0.80
5.38 $\times \;10^{-2}$	8.20 $\times \;10^{-2}$	0.90	8.32	−0.60	7.84 $\times \;10^{-2}$	0.45	5.41	−0.97
2.67 $\times \;10^{-2}$	2.75 $\times \;10^{-2}$	1.56	5.94	0.48	4.64 $\times \;10^{-2}$	0.75	4.66	0.22
1.32 $\times \;10^{-2}$	2.78 $\times \;10^{-3}$	3.27	1.57	1.90	2.41 $\times \;10^{-2}$	0.94	2.65	0.81

## Data Availability

Data are contained within the article.
